# Tincture of Iodine in the Treatment of Erysipelas

**Published:** 1911-10

**Authors:** 


### Tincture of Iodine in the Treatment of Erysipelas.

The author reports a series of about forty cases treated locally with iodine tincture. He considers it superior to other agents, but specifies certain points in the technique which are essential to success. The zone of sound skin surrounding the involved area is first painted with a wad of sterile cotton dipped in the tincture; next the diseased area is painted, using a fresh wad; finally, the area is covered over with cotton, to prevent spreading of the infection through the intermediary of the patient's fingers. The author found it best to apply the iodine lightly five or six times a day rather than more freely morning and evening. In this way induration of the superficial skin layers, which would interfere with the action of the iodine, is avoided.

The tincture used should be of 10 to 12 per cent strength, and freshly prepared.

Twenty-one cases of facial erysipelas were thus treated with uniform success, 1 recovering after the first application, 16 in three days, 3 in four days, and 1 in five days. Of 6 cases of erysipelas of the neck, 2 were relieved in four days and 4 in six days. Eleven cases of erysipelas complicating accidental wounds yielded in less than five days. The wounds were left unsutured and treated likewise with iodine, with good results.—Ferrari, in *The Gazzetta degli Ospedali e delle Cliniche.—Charlotte Medical Journal.*

---

CIMICIFUGA FOR AFTER-PAINS.—After-pains are frequently relieved by small doses of cimicifuga, especially in those cases which seem to be kept up by a neuralgic disposition or a mental and nervous irritability, and the patient is sleepless, restless, sensitive and low-spirited.—*Medical Summary.*

---

FOR REMOVING PLASTER.—According to Dr. E. J. G. Beardsley, adhesive plaster may be easily and quickly removed from the skin by the application of oil of wintergreen. When extensive areas of plaster are to be removed, an ointment of lanolin, containing 10 per cent of oil of wintergreen is better.—*Medical Brief.*

---

Smearing vaseline over the buttocks in a rectal examination, scratching the furniture with basins, spattering the carpet with plaster of Paris, are some of the "little things" that will lead some patients to consult thereafter surgeons with more neatness, if less skill.—*American Journal of Surgery.*

Don't jump to the conclusion that a benign stricture of the rectum is syphilitic. Gonorrheal proctitis causes dense cicatricial infiltration.—*American Journal of Surgery.*

---

When dealing with a chronic bone abscess (and often, too, in acuter cases) it saves weeks of after-treatment to work with an osteo-periosteal ("coffin-lid") flap, and close the wound completely.—*American Journal of Surgery.*

---

A sharp pain felt at the outer end of the groin upon sudden motion of the thigh, as in starting forward from a crouching position in a foot race, suggests fracture of the anterior spine of the ilium. This occurs usually in adolescents.—*American Journal of Surgery.*

